# PD213 An Unmet Health-Related Needs Evidence Database For Informing Policy And Innovation Decisions In Health Care

**DOI:** 10.1017/S0266462324004343

**Published:** 2025-01-07

**Authors:** Irina Cleemput, Muriel Levy, Claudia Schönborn, Mats De Jaeger, Laurence Kohn, Rani Claerman, Robby De Pauw, Charline Maertens de Noordhout

## Abstract

**Introduction:**

As there is no standard definition of unmet patient or societal needs, the Needs Examination, Evaluation, Dissemination (NEED) project defined criteria for assessing these needs. This study describes the creation of an exploitable evidence database on unmet health-related patient and societal needs for various health conditions, and how this can be used in decision-making.

**Methods:**

The NEED framework defines explicit dimensions (patient, societal, and future needs), domains (health, healthcare, and social needs), and criteria, along with specific measurable indicators, to identify unmet health-related patient and societal needs for various health conditions. For each indicator, information sources were sought, including both quantitative and qualitative evidence. For some indicators, guidance on evidence generation was developed. Possible uses of the NEED database were discussed with national and international panels of experts, stakeholders, and decision-makers.

**Results:**

Data sources were identified for some but not all patient and societal needs criteria. They included existing databases (e.g., the Global Burden of Disease database and administrative databases), literature, and primary data collection. A standardized methodology was defined for primary data collection (quantitative and qualitative). The NEED evidence database can be used by researchers, research funders, regulators, health technology assessment agencies, policymakers, patients, healthcare providers, etcetera for prioritizing areas of research as well as—thanks to the level of detail provided in the database—to assess the extent to which proposed “solutions” meet the most pressing unmet needs of patients or society.

**Conclusions:**

Once operational, an unmet patient and societal needs evidence database, anchored to an explicit framework, can serve different stakeholders and decision-makers for various purposes. It collects evidence on unmet health-related patient and societal needs for various health conditions, given the current standard of care, independent of any new product or service, and therefore supports a needs-driven healthcare innovation and policy system.

